# Experimental and theoretical investigation of acetamiprid adsorption on nano carbons and novel PVC membrane electrode for acetamiprid measurement

**DOI:** 10.1038/s41598-022-16459-x

**Published:** 2022-07-15

**Authors:** Razieh Razavi, Moslem Basij, Hadi Beitollahi, Saleh Panahandeh

**Affiliations:** 1 Department of Chemistry, Faculty of Science, University of Jiroft, Jiroft, Iran; 2 Department of Plant Protection, Faculty of Agriculture, University of Jiroft, Jiroft, Iran; 3 Environment Department, Institute of Science and High Technology and Environmental Sciences, Graduate University of Advanced Technology, Kerman, Iran; 4 Department of Plant Protection, Faculty of Agriculture, Shahid Bahonar University of Kerman, Kerman, Iran

**Keywords:** Developmental biology, Chemistry, Materials science, Nanoscience and technology

## Abstract

Acetamiprid removal was investigated by synthesized Graphene oxide, multiwall nanotube and graphite from an aqueous solution. For this propose, FT-IR, XRD, UV–Vis, SEM and EDS were used to characterize the synthesized nano adsorbents and to determine the removal process. A novel PVC membrane electrode as selective electrode made for determining the concentration of acetamiprid. Batch adsorption studies were conducted to investigate the effect of temperature, initial acetamiprid concentration, adsorbent type and contact time as important adsorption parameters. The maximum equilibrium time was found to be 15 min for graphene oxide. The kinetics studies showed that the adsorption of acetamiprid followed the pseudo-second-order kinetics mechnism. All the adsorption equilibrium data were well fitted to the Langmuir isotherm model and maximum monolayer adsorption capacity 99 percent. Docking data of adsorption have resulted in the same as experimental data in good manner and confirmed the adsorption process.

## Introduction

Insecticides or pesticides have currently led to significant challenges concerning the safety issues and contamination of foods. Careless consumption of such compounds regardless of controlling or optimizing their concentrations may threaten humans’ lives and health due to their toxic nature. Unfortunately, pesticides are used both in agriculture and non-agriculture fields^1^, including the control of insects and vector-borne diseases in residential as well as industrial areas. Therefore, there is serious need for unsophisticated, cheap, and valid procedures aimed at monitoring and detecting the pesticide residuals in different food productions^2–5^.

The design of new members of these compounds has led to promising results in recent years. In fact, the nitro group fixation in a cis configuration for different neonicotinoid types has yielded compounds with certain biologic functions and behaviors, supporting their potential efficiency to elucidate the pharmacology and characteristics of the neonicotinoid binding sites. Neonicotinoids consist of 5-membered ring derivatives, including imidacloprid and thiacloprid, 6-membered ring compounds, including thiamethoxam, and also noncyclic structures such as nitenpyram, acetamiprid^6–11^, clothianidin, and dinotefuran employed for a wide range of pests^12–14^. Organophosphates are becoming more and more common as pesticides across the world and have recently replaced organochlorine insecticide^15^. As chemical pesticides control the pests and different diseases, they play a significant role in increasing agricultural yield while helping in the prevention of diseases brought by insects, such as malaria, dengue, encephalitis, filariasis, and so on in the field of human health^16^.

Pesticides are mainly regarded as chemical mutagens. Pesticide elimination from the water system has currently become an important environmental issue. This is because, the residuals of these compounds have entered into underground water, resulting in serious concerns due to their significant growth in recent years. Conducting research to find out a unique approach aimed at removing these harmful chemicals worldwide is very challenging due to the extensive scope of pesticides used globally. There are a variety of techniques to remove pesticides, including photocatalytic degradation^17,18^, combined photo-Fenton and biological oxidation^19^, developed oxidation procedures^20,21^, aerobic degradation^22–24^, nanofiltration membranes^25^, ozonation^26^, coagulation^27,28^, fluid extraction^29,30^, solid phase extraction^31^, well as adsorption^32–36^. Kyriakopoulos and Doulia^37^ examined pesticide adsorption on carbonaceous^38^ and polymeric^39^ substances from aqueous solutions. In another study, Ahmad et al. and Hameed^40^ examined pesticide residuals detoxification using activated carbon. Adsorption can be utilized to decontaminate water as it is a popular equilibrium separation procedure and performs effectively in this regard^16^.

The current developments in carbon materials have led to graphene as a fundamental component of graphitic materials of all other ranges, representing a single atomic layer of graphite with hexagonally bonded and sp^2^ hybridized carbons^41^. Scientists and researchers have been interested in this material since it was discovered in 2004, because it showed specific characteristics, including considerable quantum Hall impacts, high mobility, great electronic^42^ and mechanical properties^43^, specific magnetism and high thermal conductivity^44^, There have been investigations on the use of graphene-based materials to manage the environmental issues related to pollution^45^.

In this study, acetamiprid removal was investigated by three different nano carbon materials. Acetamiprid adsorption with the use of GO, multi-wall nanotubes, and graphite has not been previously examined through comparative studies. However, investigations revealed that time, temperature and acetamiprid concentration variations affected the adsorbents. The study also conducted the theoretical calculations.

## Methods and materials

The materials used in this study included reagent grade dioctyl phthalate (DOP), dibutyl phthalate (DBP), dimethyl sebacate (DMS), 2-nitrophenyloctyl ether (NPOE), tetrahydrofuran (THF) and high molecular weight PVC (provided by Merck). The study used highly pure sodium tetraphenyl borate (NaTPB), NaNO_3_, MWNTC, graphite, potassium permanganate, Peroxide hydrogen, sulfuric acid, (provided by Merck), which means that they did not need any additional purifications. Technical materials of acetamiprid, with a purity level higher than 95%, were obtained from Giah Company, Iran.

### Graphene oxide (GO) synthesis

Modified Hummers’ technique with slight changes^46,47^. was utilized to synthesize graphene oxide. The addition of graphite powder (3.2 g) and NaNO_3_ (3.2 g) into 98% H_2_SO_4_ (140 mL) took place in an Erlenmeyer flask (800 mL), in an ice-bath (˂ 10 °C) while constantly stirred. Gradual addition of KMnO_4_ (18.5 g) to the suspension was done after the compound was stirred for a four-hour period in this temperature, after which the suspension was stirred for another one hour at a temperature of < 15 °C. Then, the gradual addition of 300 mL distilled water to the crude mixture was followed by stirring the mixture for another two hours, raising the temperature to 35 °C with constantly stirring for 2 h. In the next step, addition of 60 mL of a H_2_O_2_(30%) aqueous solution led to changes in the color of the mixture from dark brown into yellow. The obtained compound underwent centrifugation at 4400 rpm over a 20-min period, followed by its frequent washing by 5% HCl solution and deionized water to reach a gel-like substance with neutral value. The obtained substance was centrifuged and then vacuum dried at a temperature of 70 °C to produce the powder of graphene oxide.

### Analysis of absorbance

The present research carried out acetamiprid equilibrium adsorption on graphene oxide, multi-walled nanotubes, and graphite in polyethylene tubes. The study also considered constant concentrations of acetamiprid in different time and temperature conditions with the use of an orbital shaker which operated at 150 rpm agitation speed. It also utilized 10 ppm stock solutions of acetamiprid. Analysis of the solution for concentrations of acetamiprid and its adsorption on the adsorbent at equilibrium time with the use of UV–Visible spectroscopy were conducted following magnetic separation and filtration. The relation below was used to calculate the acetamiprid adsorption on the adsorbent at equilibrium time^48,49^ of qe:

1 $${q}_{e=\frac{V({C}_{0}-{C}_{e })}{m}}$$

In which, Co, Ce, V, and m represent the primary acetamiprid concentrations (mg/L), acetamiprid concentrations (mg/L) in solution following the adsorption, the solution volume (L), and the mass (g) of graphene oxide, multi-walled nanotubes, and graphite, respectively. the following relation was used to calculate the percentage of acetamiprid removal:

2 $$\left(\%R\right)=100\times \frac{{C}_{0 }-{C}_{t}}{{C}_{0}}$$

In which, Co and Ct indicate the primary acetamiprid solution concentrations (mg/L) and the ultimate concentrations (mg/L) following the process of adsorption, respectively. Relation 3 was used to calculate adsorption capacity from the adsorbent mass and acetamiprid solution volume:

3 $${q}_{t}\left(mg\right)=V\times \frac{{C}_{0 }-{C}_{t}}{m}$$

In which m and V represent the adsorbent dose (mg) and acetamiprid solution volume (L).

### PVC membrane

Preparation of the membrane electrode took place according to previous reports^50,51^. Dissolution of the sensor material, with acetophenone (0.002 g) acting as a plasticizer, and PVC (0.004 g) was done in 2 ml THF. NaTPB (0.001 g) played the role of an additive in several cases and graphite, graphene oxide and multi-wall nanotube (0.002 g) as ionphore. To obtain an oily concentrated compound, slow evaporation of the THF solution was conducted at ambient temperature. Formation of a transparent membrane with a thickness of around 0.3 mm resulted from dipping a Pyrex tube (3–5 mm i.d.) into the obtained compound for an approximately 10-s period. After pulling the tube out of the compound and keeping it at ambient temperature for a 1-h interval, internal filling solution (1.0 × 10^−2^ M acetamiprid) was used to fill it. Ultimately, electrode conditioning was obtained as it was soaked in a 0.1 M acetamiprid solution for a 36-h period.

### Potentiometery technique

The study applied a cell assembly of the type described below: Ag/AgCl | 10^−2^ M acetamiprid | sensor membrane | sample solution | reference electrode. A saturated calomel electrode with an Orion digital research pH-mV meter were considered to make the emf observations. The emf of acetamiprid solutions whose concentrations ranged from 10^−1^ to 10^−9^ M by serial dilution was measured to investigate the electrode performance. Stirring of the solutions and recording of the reading potentials after reaching stability were performed in the next steps, followed by plotting as a logarithmic function of acetamiprid activities, based on γ as the activity coefficient. Debye–Hückel relation^52^, which can be also applied to acetamiprid was used for data calculation:

4 $$log\gamma =-0.511{Z}^{2 }[\frac{{\mu }^\frac{1}{2}}{1+1.5{\mu }^\frac{1}{2}}-0.2\mu ]$$

In which, µ represents the ionic strength and Z is the valency. The temperature of 25 ± 0.1 °C was considered to carry out all measurements. Fresh preparation of the acetamid solutions took place using precise dilution from their stock standard solution of 0.1 M, with distilled, de-ionized water.

### Computational methods

The initial step was allocated to full geometric optimization of acetamiprid molecules, carbon nanotubes, graphene oxide, as well as graphite using density functional theory (B3LYP and basis set 6-311G(d)), by drawing 3D structures by guass view 5 software, determining the energy groups and composed molecules of each. The ArgusLab molecular modeling software was used for a quest of conformations, after which the molecular adsorption energies, density of states, ESP, and chemical parameters could be calculated.

## Result and discussion

### characterizing synthesized graphene oxide (GO)

#### IR analysis

**Figure 1:**
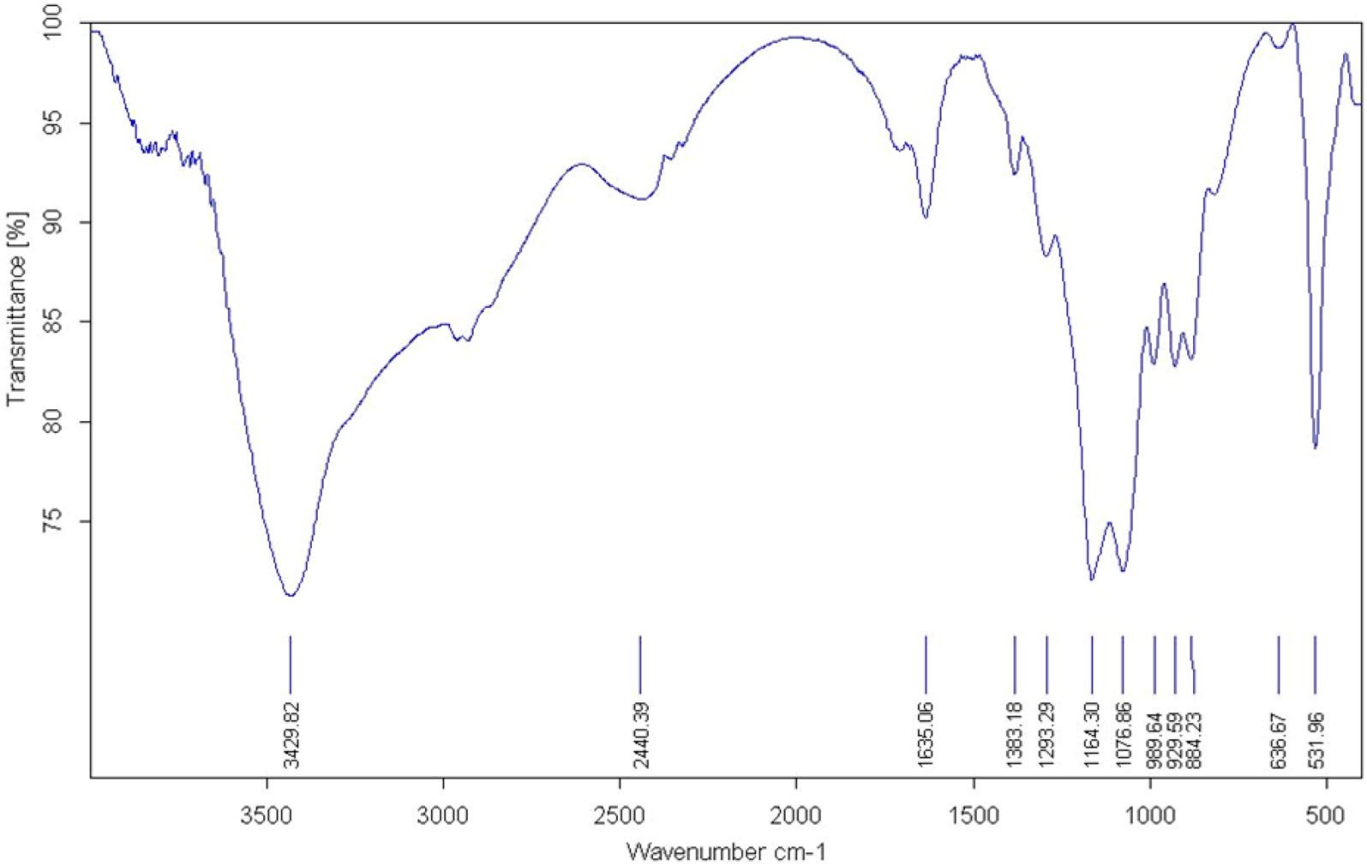
IR spectrum of synthesized graphene oxide.

Hummer method was used for synthesis of graphene oxide**,** the functionality of which was supported by Fourier transform infrared spectroscopy. According to Fig. 1, there is a wide peak at 3429 cm^−1^ in the area with high frequency due to O–H bond stretching mode, revealing that hydroxyl groups are present in GO. The carboxyl group had a band at 1720 cm^−1^. The stretching and bending vibrations of OH groups found in water molecules adsorbed on GO resulted in a dramatic or resonance peak at 1615 cm^−1^. The peaks at 1356, 1225, and 1056 cm^−1^ denote the C–OH group, C–O–C stretching, and the C–O group vibration mode, respectively.

#### XRD analysis

**Figure 2:**
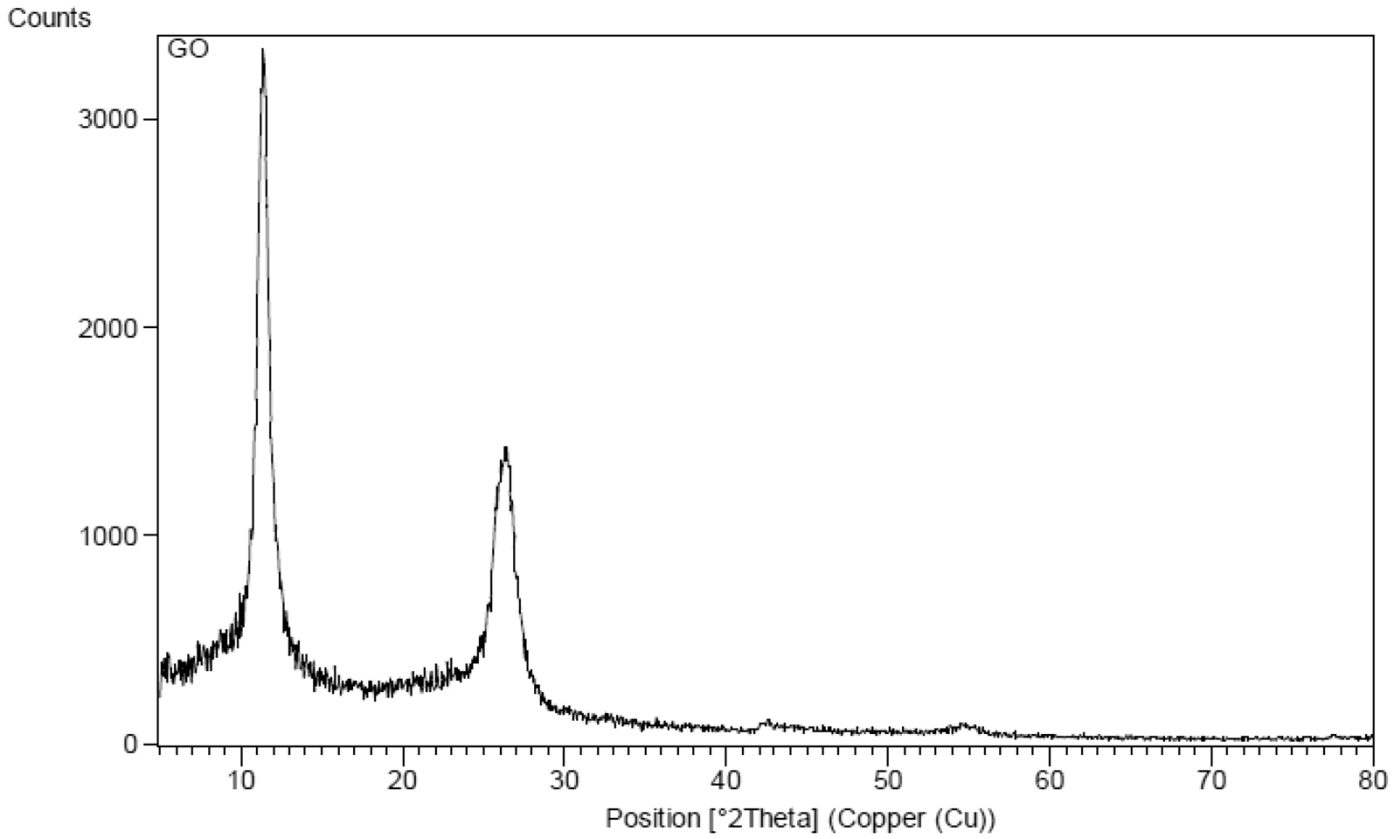
XRD patterns for the synthesized graphene oxide.

The crystalline characteristics and phase purity of the synthesized graphene oxide are characterized through XRD analyses as shown in Fig. 2. Intense and dramatic differentiation peaks are observed for pure graphite at 2θ = 26.55°, corresponding to the (002) plane of hexagonal graphite structure with 0.34 nm interlayer spacing. Chemical oxidation and exfoliation into graphene oxide led to a decrease in 26.55° peak while resulting in a wider differentiation peak at 11.78°, indicating a 0.83-nm *d*-spacing. The increasing interlayer distance among the successive carbon basal planes shows associations with the intercalation of oxygen functional groups and water molecules into the structure of carbon layers^53^.

#### SEM analysis

**Figure 3:**
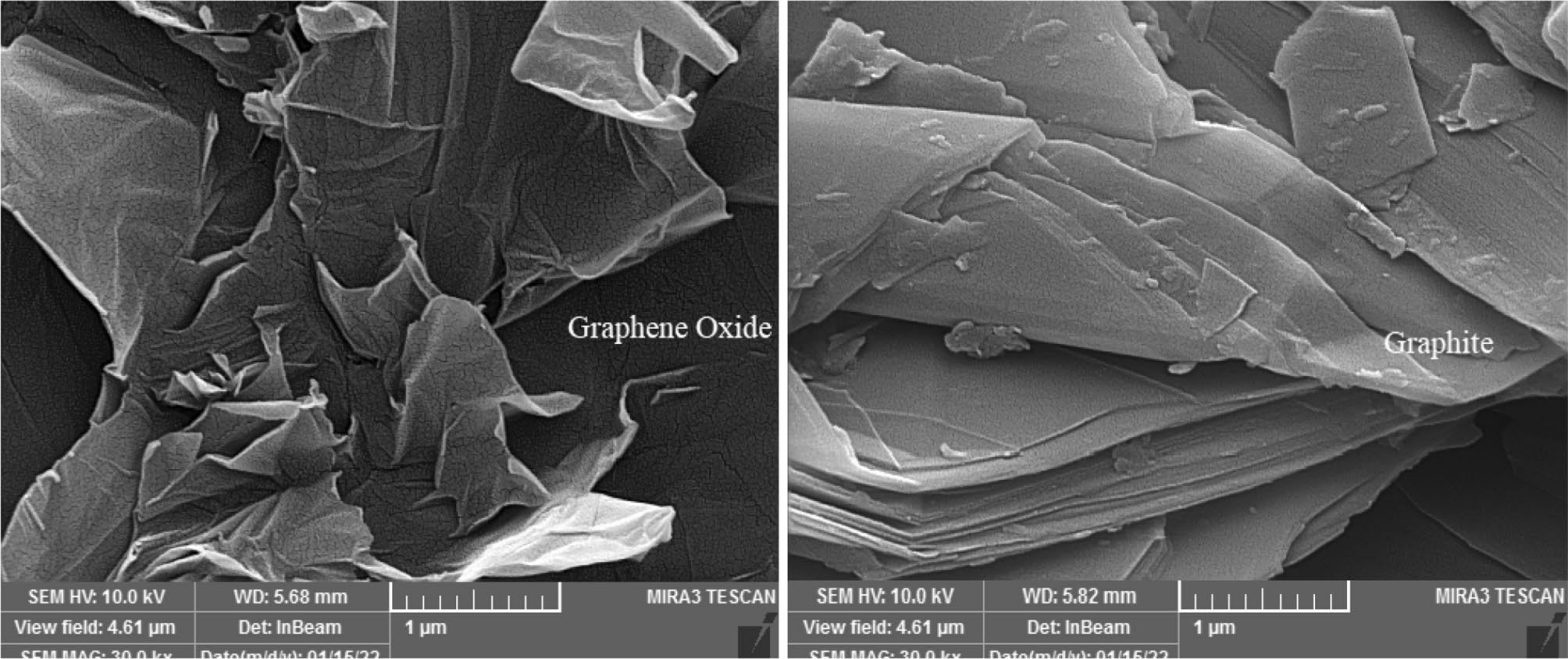
SEM of graphite and graphene oxide surface.

Figure 3 showed the changing of morphology surface of graphite after oxidation reaction to synthesis of graphene oxide. Breaking of layers in graphite is clear on SEM picture. The images prove that during graphite oxidation the number of layers reduces, crystallinity decreases and amorphization occurs. images show that the obtained GO is a single-layer product and contains “more defective”.

### Adsorption of acetamiprid

#### Adsorbent effect

**Figure 4:**
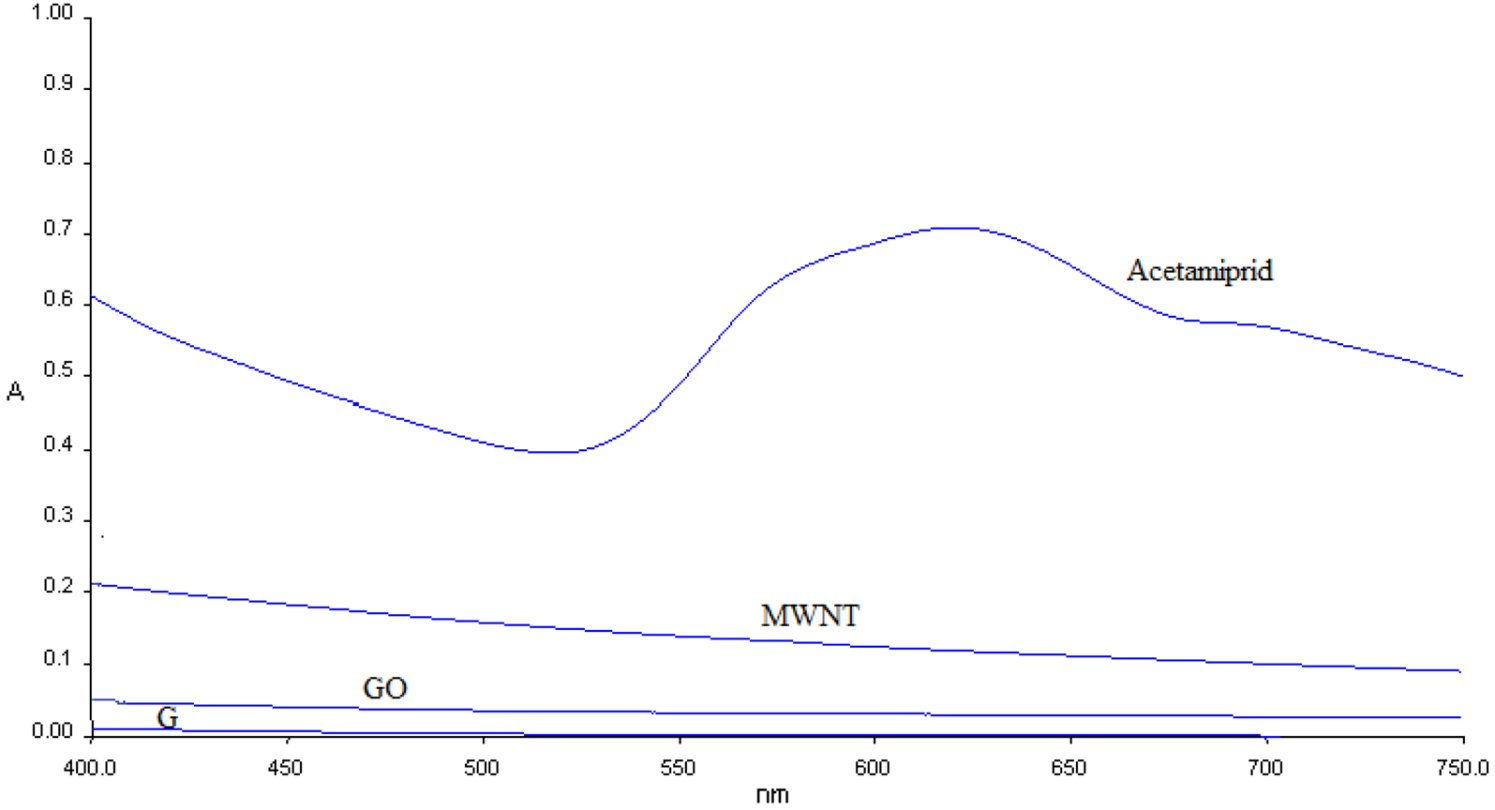
The UV spectrum of acetamiprid adsorption on graphene oxide, multi-walled nanotubes, and graphite.

The highest absorption peaks were found to be at λ_max_ 620 nm as shown by acetamiprid and acetamiprid-adsorbent UV–Vis spectra in Fig. 4, confirming successful acetamiprid adsorption on the surface of adsorbents, including graphene oxide, multi-walled nanotubes, and graphite. Acetamiprid peaks at 620 and 517 nm are associated with the n → π* transitions taking place in acetamiprid structure. The λ_max_ of acetamiprid is eliminated following adsorption at the presence of the adsorbent because of perfect omission in the solution^6^.

#### IR analyses for adsorption effects

**Figure 5:**
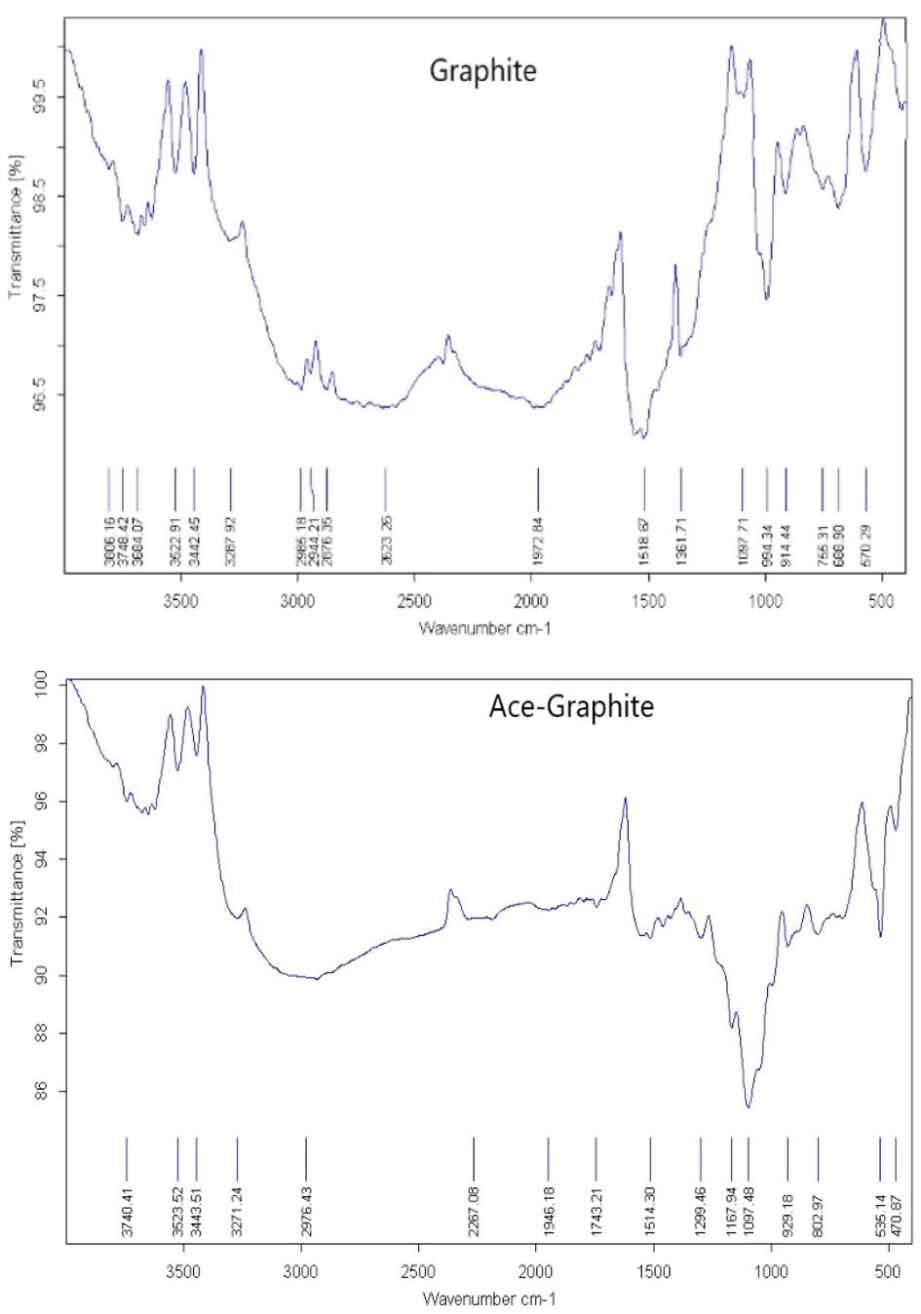
IR Spectrum of Acetamiprid-graphene.

**Figure 6:**
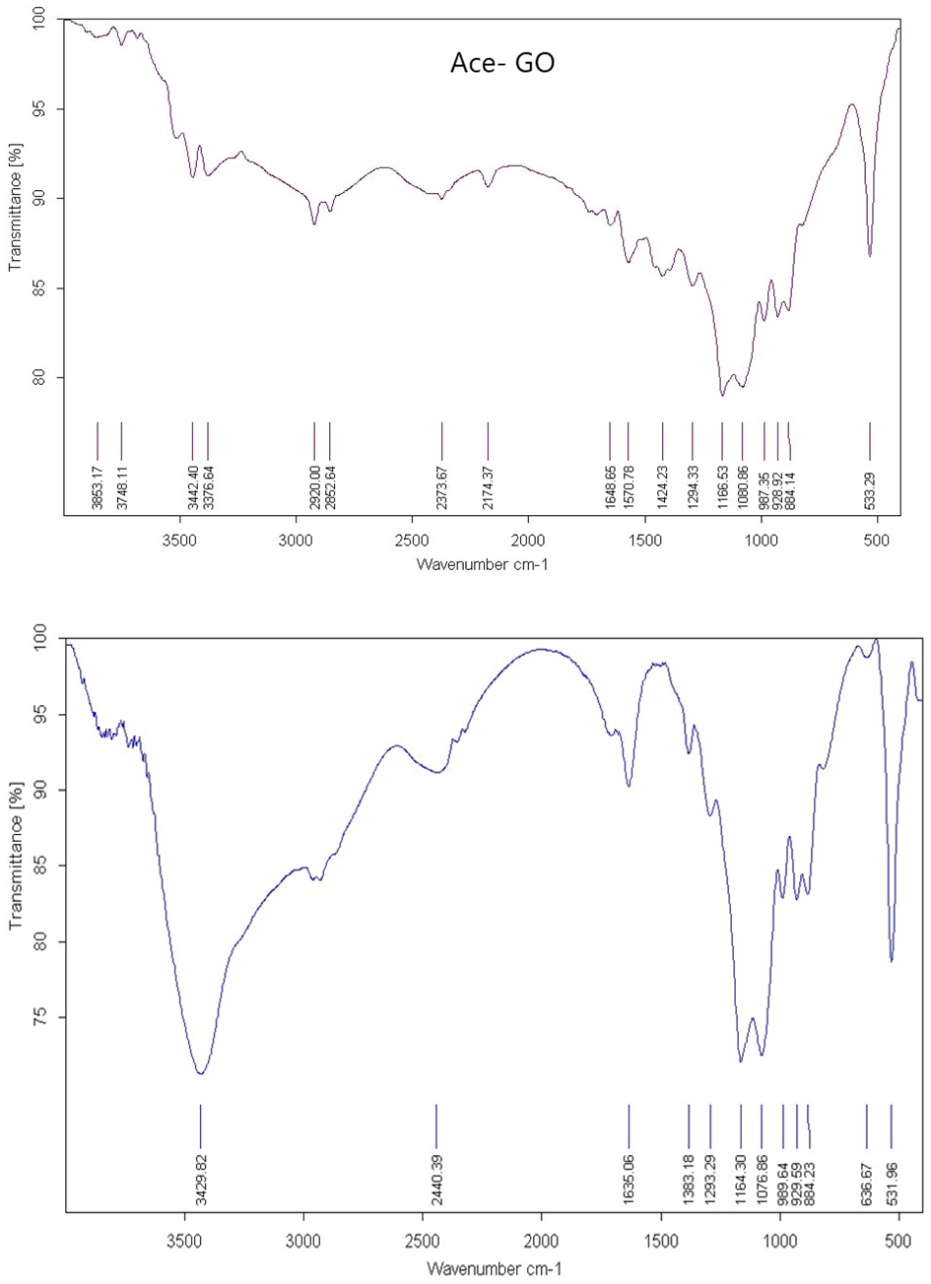
IR Spectrum of Acetamiprid-graphite oxide.

**Figure 7:**
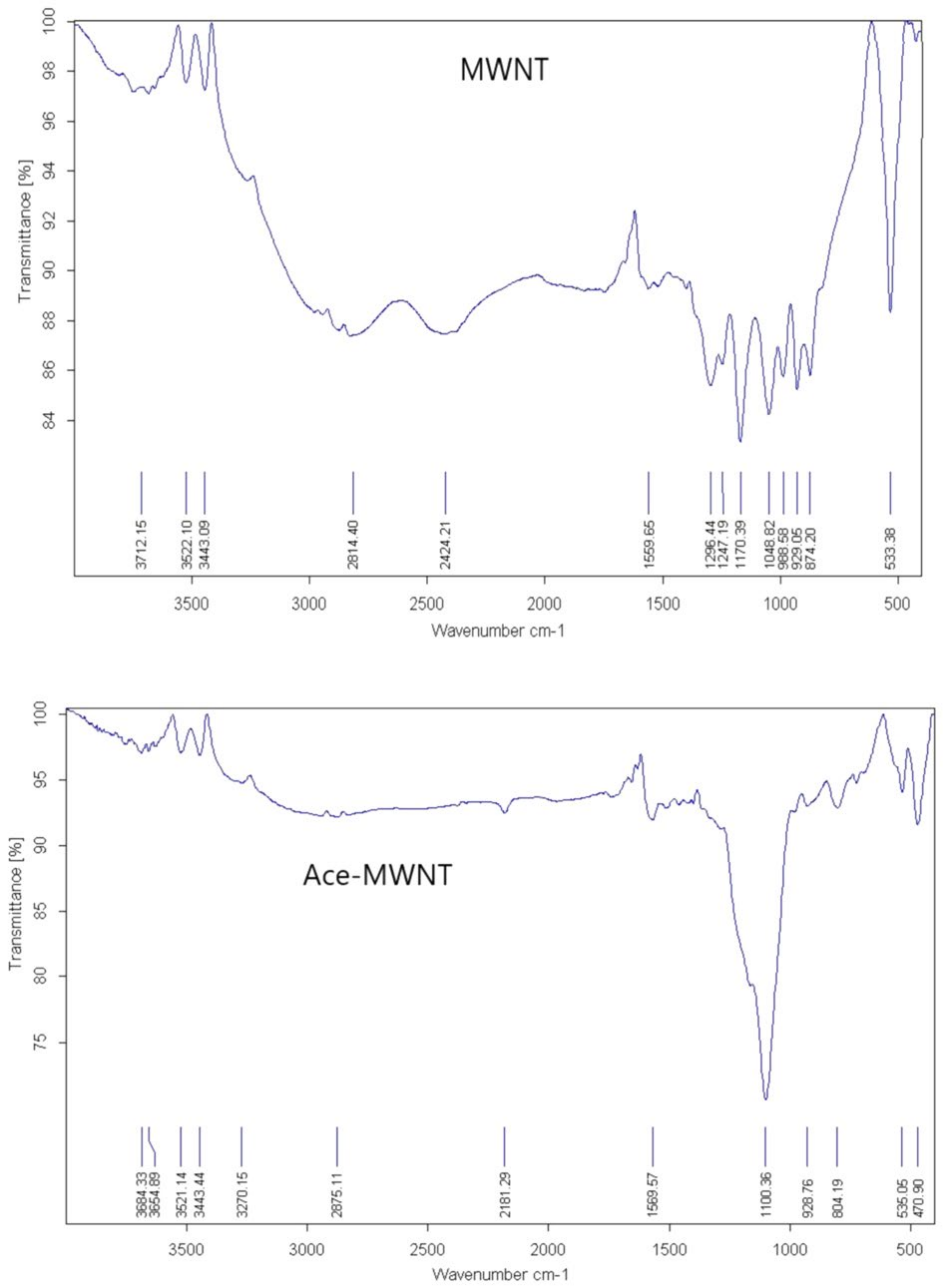
IR Spectrum of Acetamiprid- multi-walled nanotubes.

**Figure 8:**
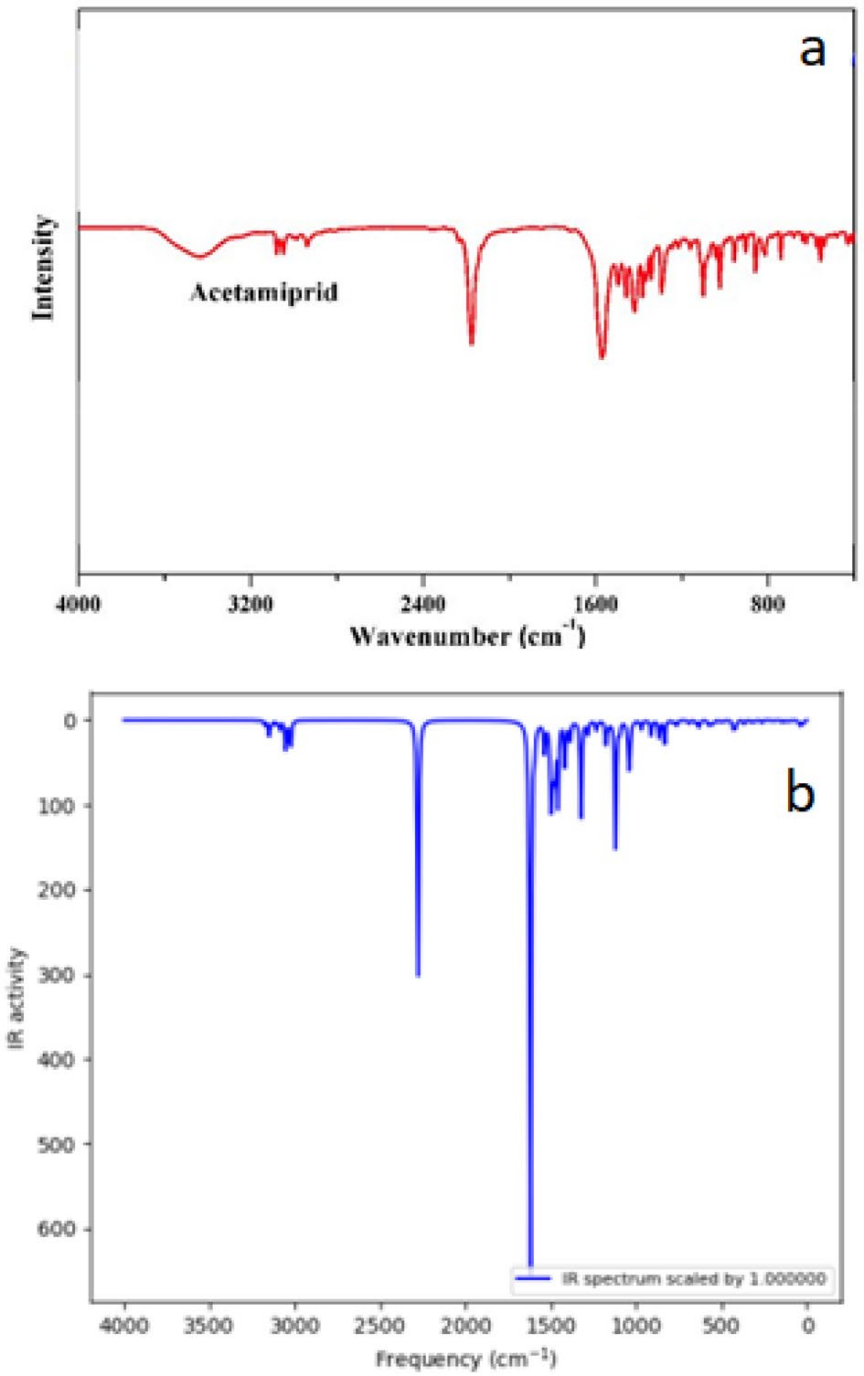
Experimental (**a**) and theoretical (**b**) acetamiprid IR spectrum.

IR spectroscopy illustrates acetamiprid adsorption on graphene oxide, multi-walled nanotubes, and graphite. As shown in Figs. 5, 6, 7 there were peaks in graphite oxide (Fig. 5.) at 3442, 1648, 1294 and 1058 cm^−1^ because of stretching vibrations of –OH, C=O (carboxyl), C–OH, and C–O. Acetamiprid^54^ had peaks at 3000 because of –CH stretching, 2240, 1640 and 1500 cm^−1^ because of CN (nitrile), C=N (Imine) and C=C aromatic, correspondingly as shown in Fig. 8a. Acetamiprid theoretical IR spectrum obtained by DFT technique calculations can be observed in Fig. 8b. Figure 5 shows complete disappearance of these bands in graphene FT-IR spectra, while there is a new peak at 3434 cm^−1^ because of graphene backbone chain skeletal vibrations. The peaks of acetamiprid were at 2976 because of –CH stretching, 2267, 1514 and 533 cm − 1 because of CN (nitrile), C=C aromatic and C–Cl^53^.

As shown in Fig. 5, there is a dramatic peak at 1100 for carbon nanotube backbone, 2876 because of –CH stretching, 2181, 1560 and 535 cm^−1^ because of CN (nitrile), C=C aromatic and C–Cl of Acetamiprid.

#### SEM analysis

**Figure 9:**
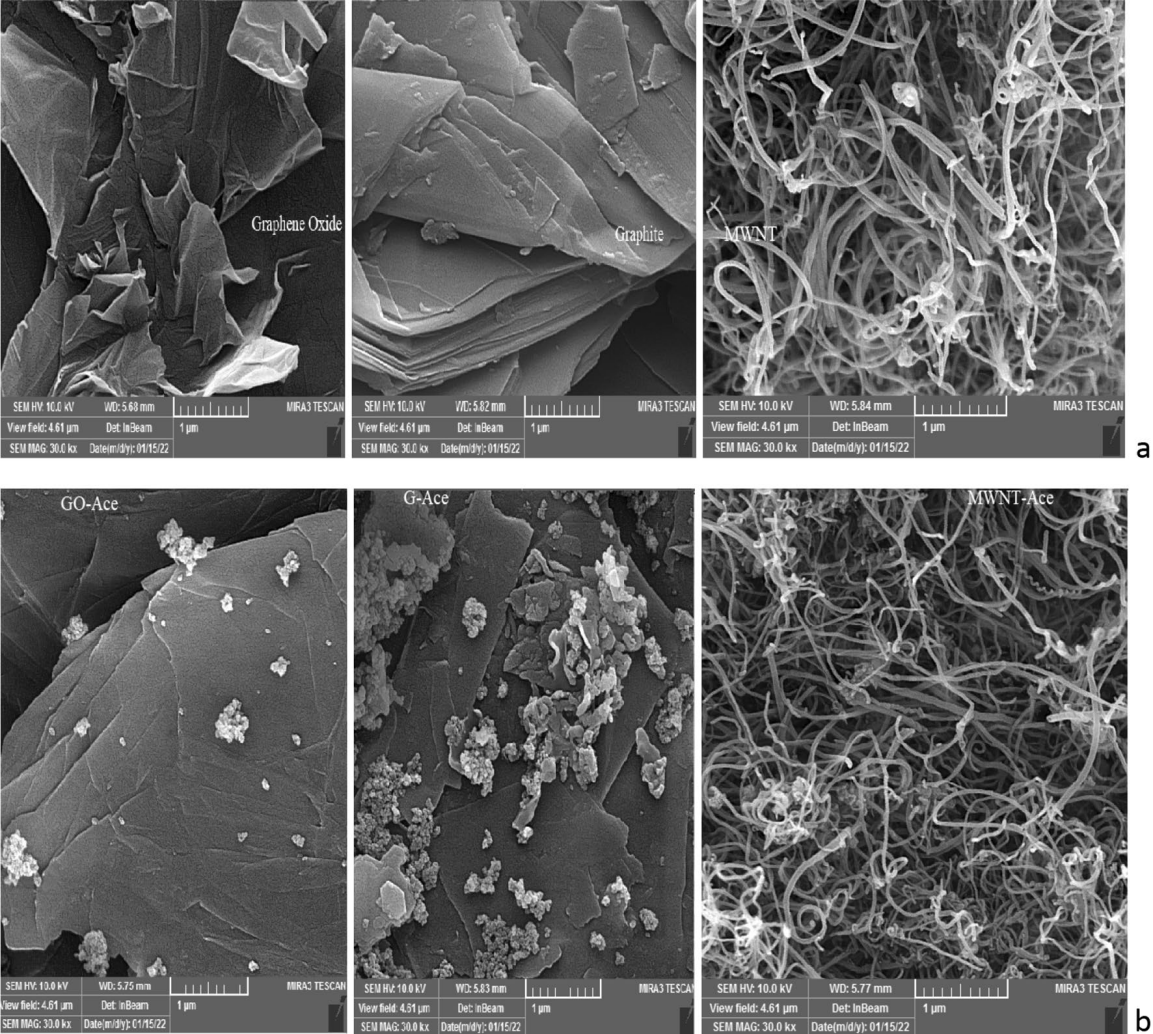
Surface of adsorbent: (**a**) before adsorption of acetamiprid (**b**) after adsorption of acetamiprid.

The surface morphologies of the adsorption materials were examined by field emission scanning electron microscopy (FE-SEM, JEOLJSM-7000F) as shown in Fig. 9a,b. SEM results for adsorption (Fig. 9b) show the aggregated and actamiprid particles on the surface of adsorbent^53^.

**Figure 10:**
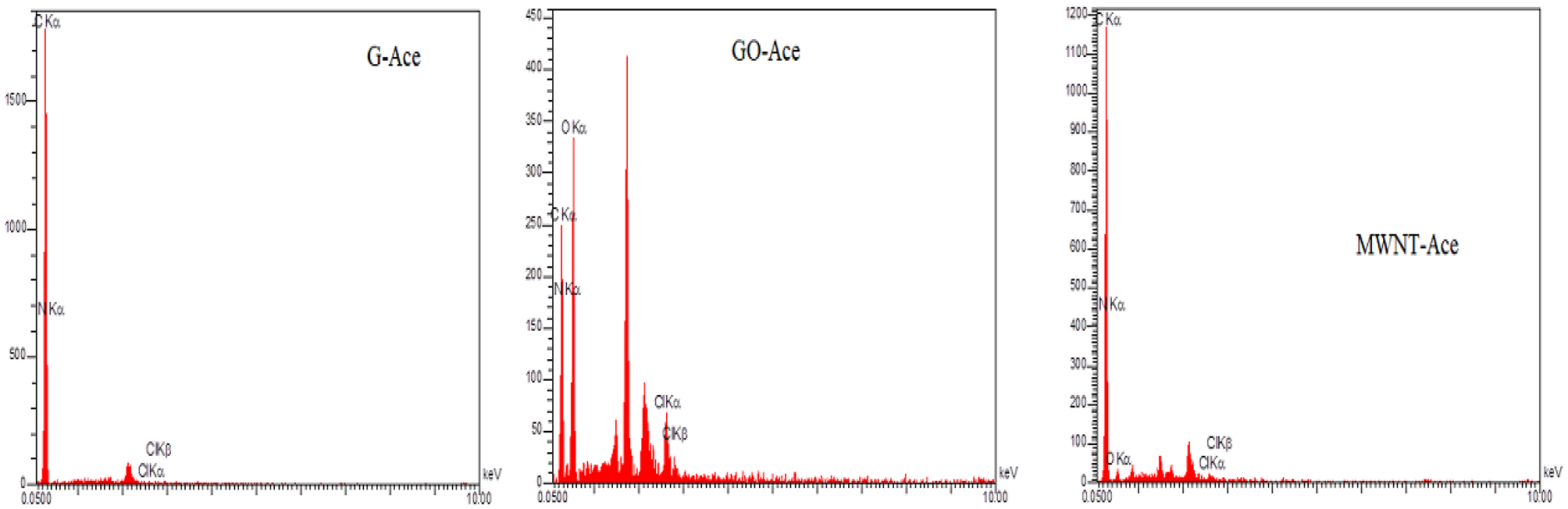
EDS of adsorption of acetamiprid.

Figure 10 depict the adsorbent@ acetamiprid composite nanoparticles EDS analysis after acetamiprid adsorption. Figure 10 used to identify the elemental composition of materials that appear in the surface of nano materials (GO, graphite and MWNT. EDS systems are attachments to Electron Microscopy instruments where the imaging capability of the microscope identifies the specimen of interest. The data generated by EDS analysis consist of spectra showing peaks corresponding to the elements making up the true composition of the sample being analyzed. Elemental mapping of a sample and image analysis are also possible. EDS curves depict peaks corresponding to N, O, C, and Cl, that refer to the acetamiprid atoms and the data obtained an intense sharp peak is only observed in the GO image where it is shown the most adsorption of acetamiprid happened on GO surface.

### Time effects

**Figure 11:**
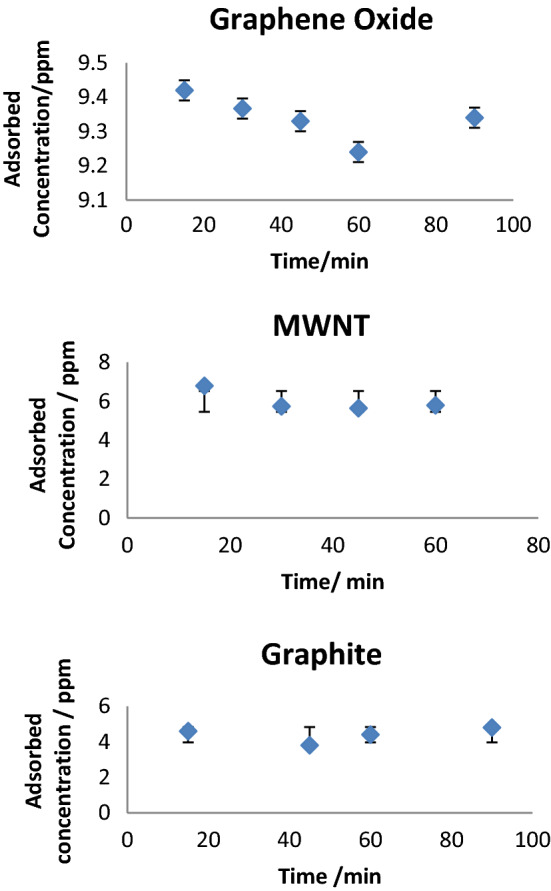
Influence of contact time on the adsorption of acetamiprid.

The study examined the contact time effects related to adsorption characteristics of graphene oxide, multi-walled nanotubes, and graphite, as shown in Fig. 11 for contact times of 15, 30, 45, 60 min to efficiently remove acetamiprid. The 60-min contact time showed the highest efficiency in removing acetamiprid; however, the 15-min contact time proved to be used for graphene oxide, multi-walled nanotubes, and graphite, resulting in surface saturation. When the contact time increased more, acetamiprid adsorption was not influenced any more due to previous saturation of the adsorption sites. Accordingly, as the contact time increased from 15 min toward 60 min, the efficiency of removal decreased, indicating that saturation prevents any more impacts of increasing time on the adsorption performance of the adsorbents.

### Temperature effects

**Figure 12:**
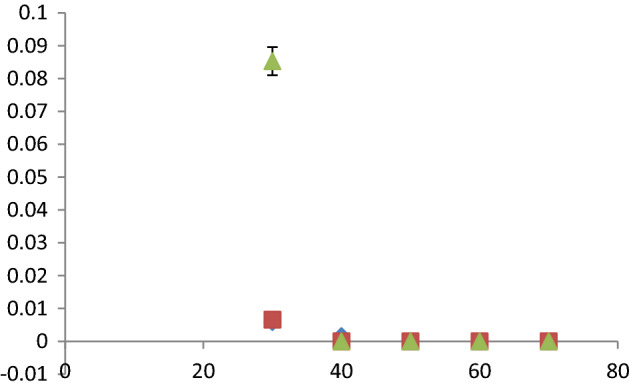
Adsorption isotherms for acetamiprid.

Application of Langmuir and Freundlich adsorption isotherms, as shown in Fig. 12, aimed at investigating the equilibrium concentrations of solutes in the adsorbent surface to solute concentrations in liquids, considering certain temperatures. The formation of monolayer adsorbates on the exterior surface of adsorbent materials is described by the Langmuir adsorption isotherm. Figure 12 shows the three adsorbents in a temperature of 25 °C with surface saturation and 100% adsorption.

### Potentiometry analysis

**Figure 13:**
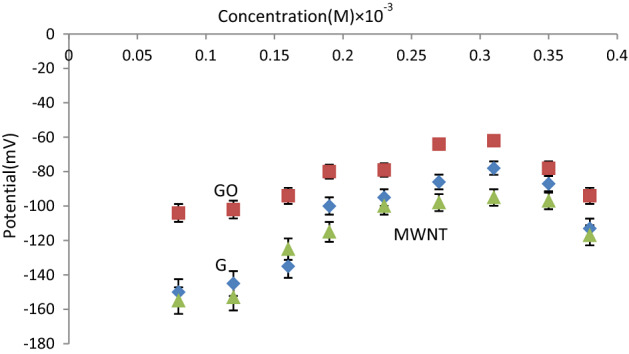
Potential responses for different PVC membrane electrodes with graphite, graphene oxide and multi-walled nanotubes.

Graphite, graphene oxide and multi-walled nanotubes are all insufficiently insoluble in aqua while having carbon atoms and heavy electron density, leading to their desirable performance as carriers in the PVC membranes concerning specific pesticides as acetamiprid of appropriate size and charge. Therefore, preliminary studies have employed it as a neutral carrier for the preparation of membrane electrodes based on PVC for acetamiprid. Figure 13 indicates the possible responses produced by electrodes with the highest sensitivity, supplied using similar empirical circumstances (with an exception for 36-h conditioning in a 1.0 × 10^−2^ M of the respective solutions). Accordingly, evidence shows that graphene oxide-based PVC membranes could appropriately determine the highest sensitivity of responses in various adsorbent experiments. Besides, EMF responses found for acetamiprid selective were considerably lower compared to Nernst equation predictions, which may be related to the selective behaviors shown in the figure for possible responses of different PVC membrane electrodes with graphite, graphene oxide, and multi-walled nanotubes. The function of graphene oxide in opposition to acetamiprid was compared with different adsorbents while examining the fast exchange kinetics resulted from graphene oxide-acetamiprid complexes. The potential responses with the required stability were produced by the ionophore PVC-based membranes (graphite, graphene oxide and multi-walled nanotubes) functioning as selective electrodes for acetamiprid. The solution used in this experiment contained 0.1 M acetamiprid following 36-h conditioning. These treatments led to Nernstian responses in various concentrations of acetamiprid solutions, while the slope was nearly fixed.

### Computational and docking analysis

**Figure 14:**
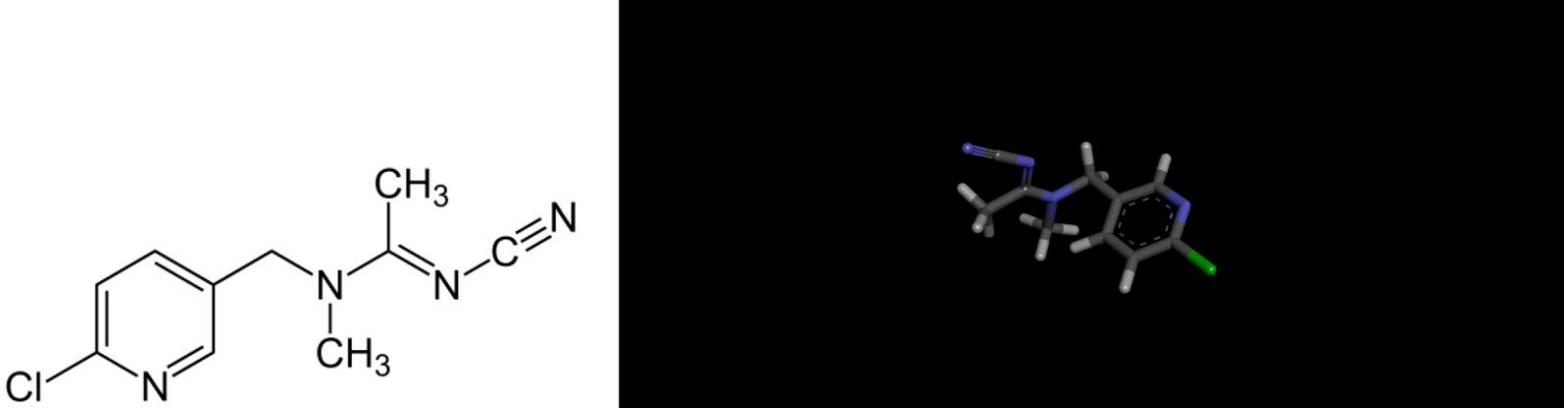
Acetamiprid Structure.

**Figure 15:**
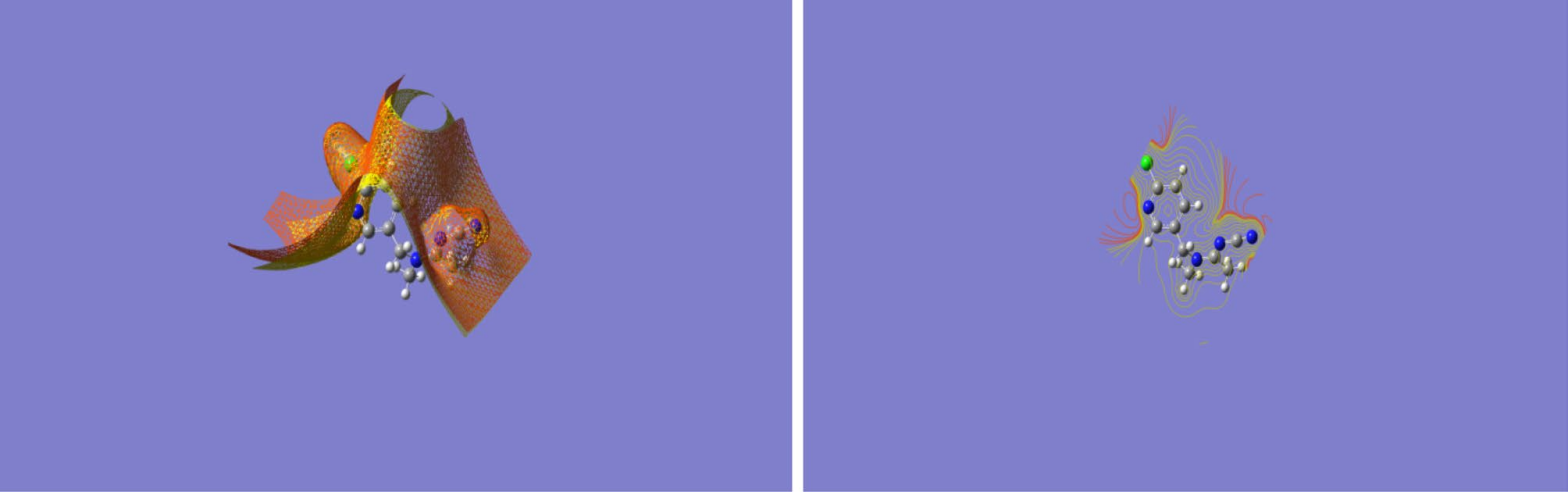
Acetamiprid electrostatic potential contours.

**Figure 16:**
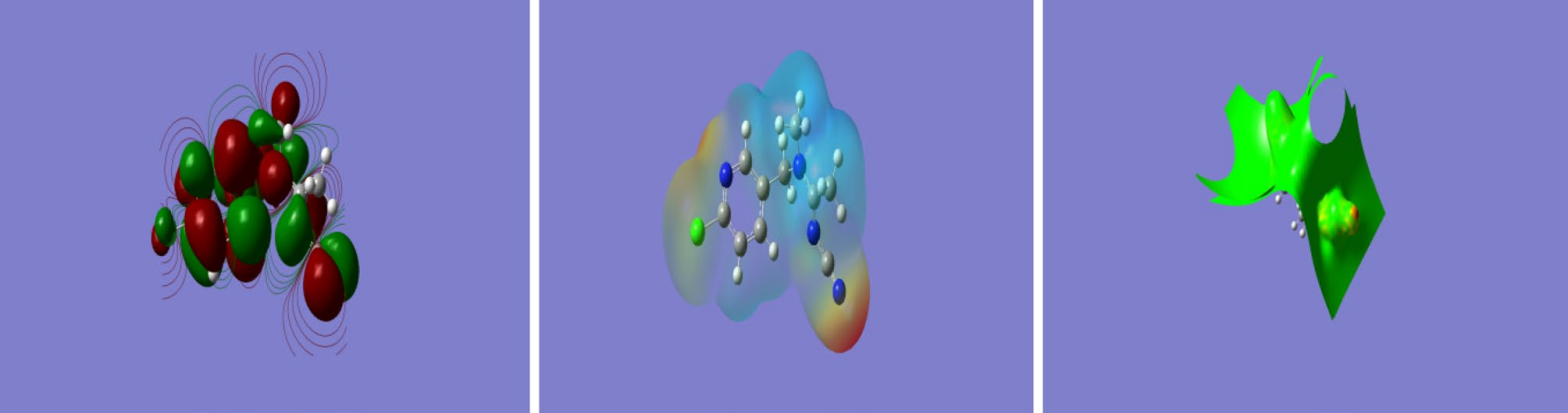
Acetamiprid electrostatic potential maps.

**Figure 17:**
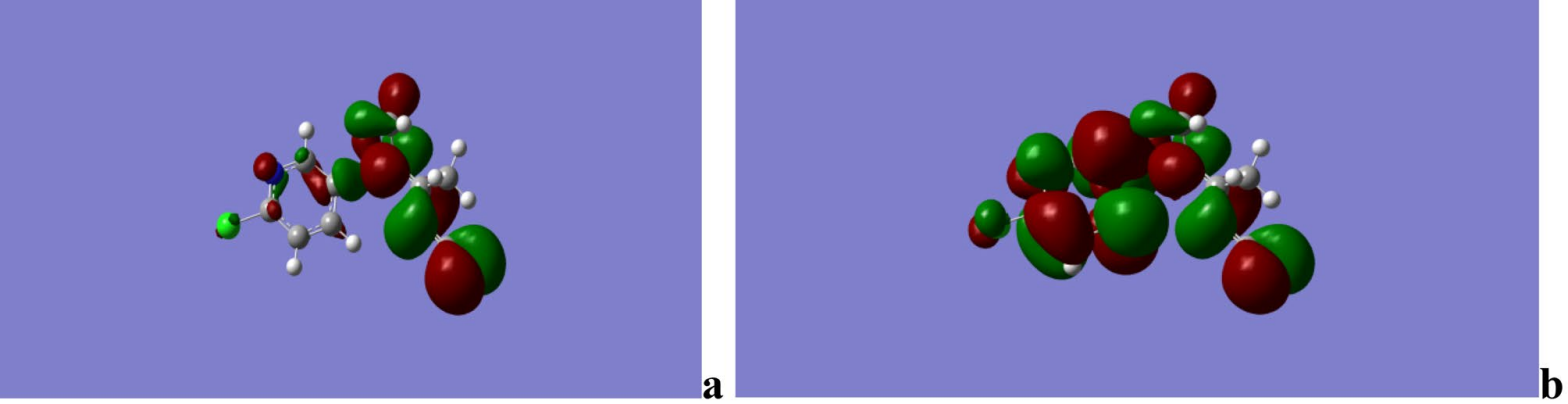
Acetamiorid HOMO (**a**) and LUMO (**b**).

A moderate, inexpensive, but valid doubly split valence basis set with polarization functions on heavy atoms: 6–311 g (d) was used to optimize and calculate the energies, enabling to accurately map n-electrons and prevent overestimation of excessive energy. Correction of fermion exchanges took place through the 3-parameter hybrid function of Becke (B3), while electron correlations were considered using the model of Lee, Yang and Parr (LYP)^55^, increased in the case of long range interaction, including hydrogen bonding through a Coulomb Attenuating Methodology while restricting electron spin. The Gaussian 09^56^ software package was used to perform the required calculations, which were carried out in the gas phase as shown in Figs. 14, 15, 16, 17, according to which the significantly negative electrostatic potential on the nitro group and the nitrile nitrogen atom of acetamiprid^24^ show their high likelihood to act as the sites for initiating interactions. Nevertheless, constructive reactions can only occur through coordination at the less electron rich *C*=*N* moiety. Thus, it can be hypothesized that adsorption initiation in neutral circumstances is carried out through coordination of adsorbent molecules with the nitrogen atoms of the *C*=*N* moiety, leading to a bonded complex and allowing close associations of the two reacting species.

The density of states (DOS) necessarily represents the number of various states at a certain level of energy occupied by the electrons, which means how many electron states can be found in each unit volume and unit energy. This function affects different bulk characteristics, including specific heat, paramagnetic susceptibility, and various transport processes associated with solids having conductivity. The overall distribution of states can be determined as an energy function using calculations of density of states, while the energy bands spacing is also determined in semi-conductors. Its mathematical representation shows distribution by a probability density function, typically representing an average across the domains of space and time in different states that the system occupies. DOS has direct associations with the dispersion relations of the system properties. Higher density of states at a certain level of energy reflects availability of a great number of states to be occupied. Continuity is generally a feature of the matter DOS. Yet, the isolated systems, like atoms or molecules in the gas phase, have discrete density distributions similar to a spectral density. Local DOS or LDOS represents local changes which are mostly because of distorted original system.

**Figure 18:**
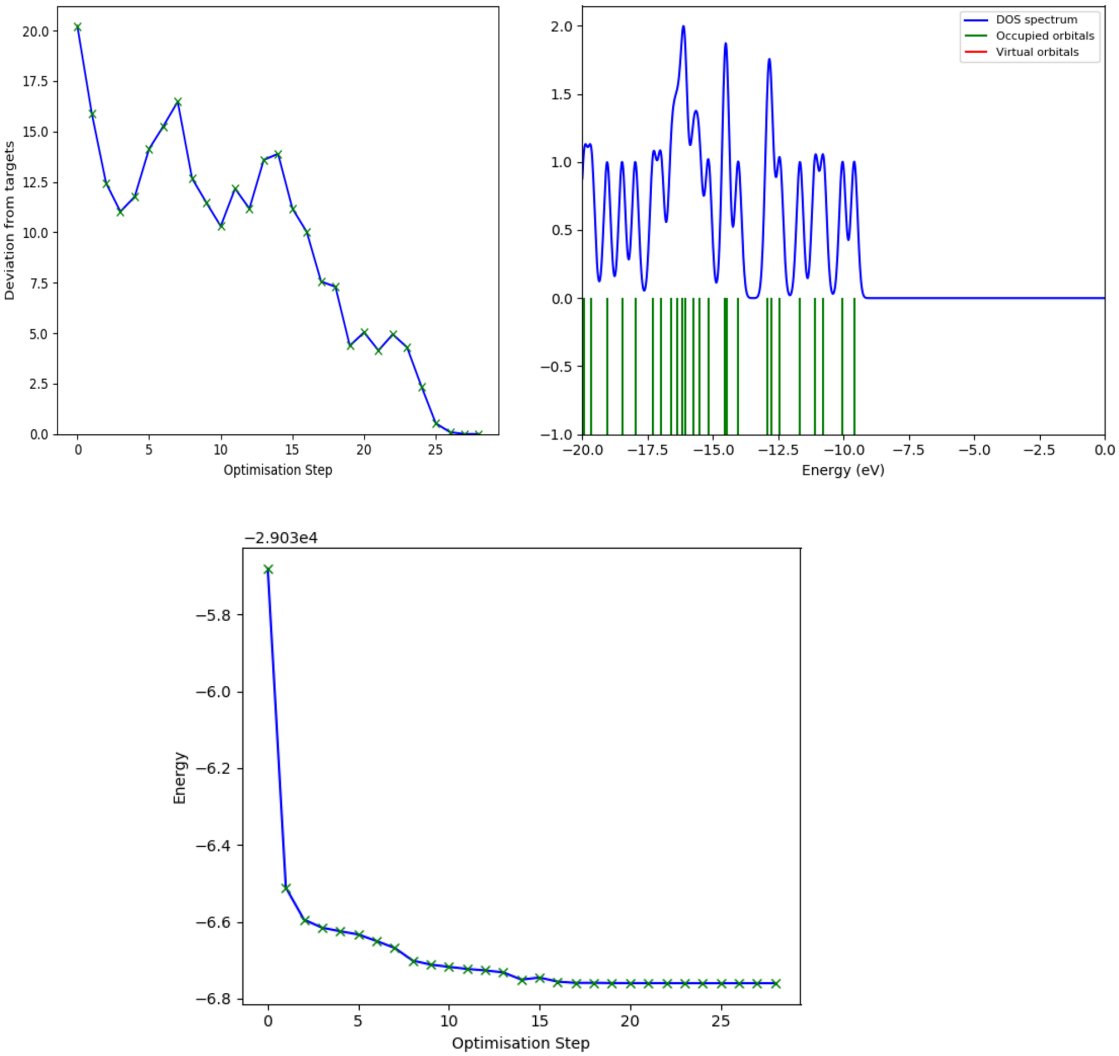
Acetamiorid DOS and Energy variations.

It is possible to calculate DOS for electrons, photons, or phonons considering the quantum mechanical system, whose results may be provided through a function of energy or the wave vector *k.* It is necessary to know the system-specific energy dispersion relationship between *E* and *k* to enable converting between the density of states as an energy or the wave vector function. Overall, the system’s typological features, including the structure of the band, affect the characteristic of DOS significantly. The 3D Euclidean topologies can be observed in the highly popular systems such as neutronium in neutron stars and free electron gases in metals (instances of degenerate matter and a Fermi gas). On the other hand, 2D Euclidean topologies are found in systems with lower popularity, including 2D electron gases of 2DEG in in graphite layers as well as the quantum Hall effect systems in devices of MOSFET type. The 1D typology can be also observed in those systems with the least popularity, including carbon nanotubes, the quantum wire, and Luttinger liquid. Given the achievements in the field of nanotechnologies and nanomaterials, the last two typologies which are less familiar have the potential of becoming more common. As shown in Fig. 18, acetamiprid represents a one-dimensional topology of quantum wire.

**Figure 19:**
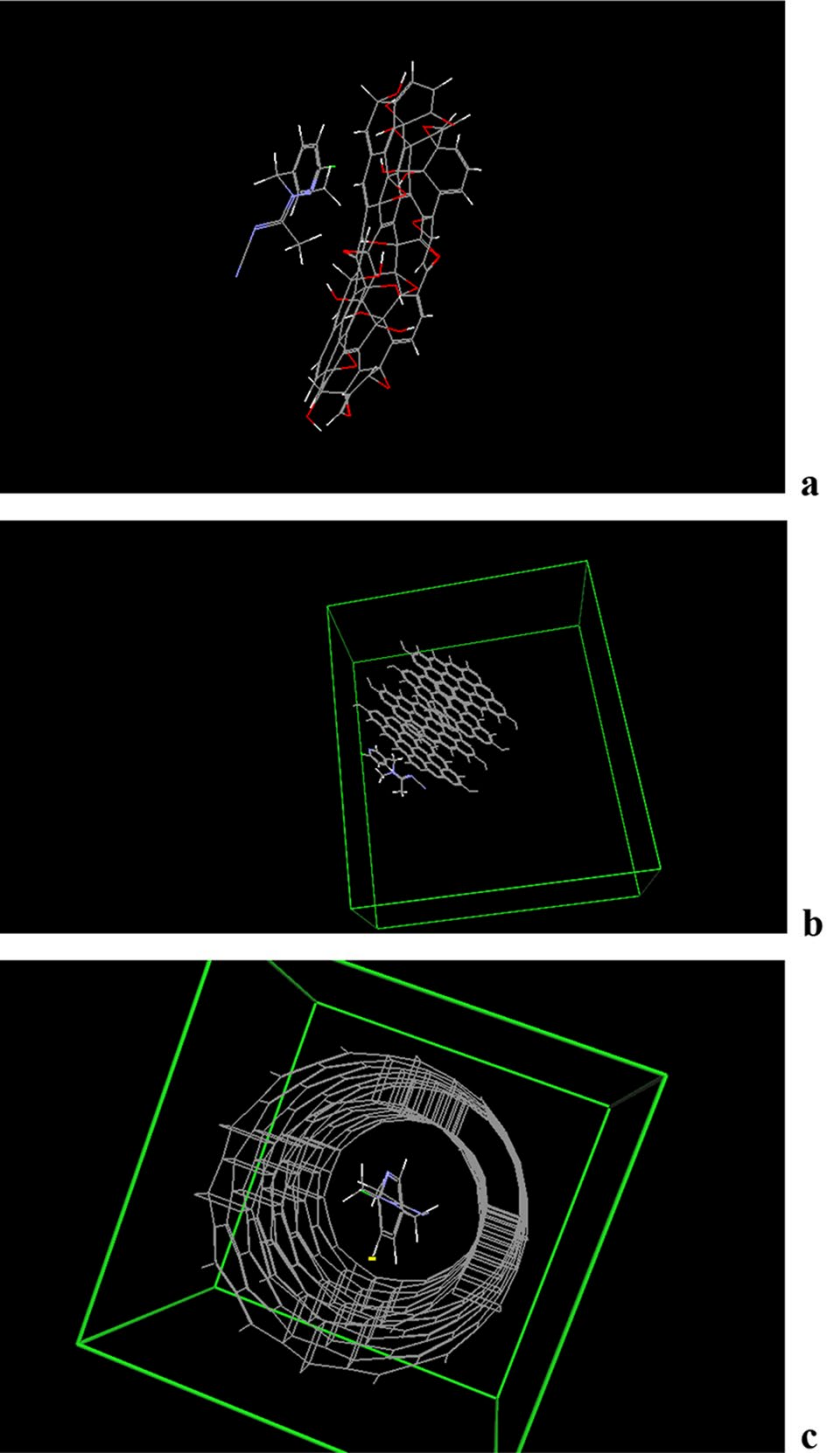
Graphene oxide (**a**), graphite (**b**) and multi-walled nanotubes (**c**) docking using acetamiprid.

Table 1 and Fig. 19 report graphene oxide (a), graphite (b) and multi-walled nanotubes (c) docking energies and complicated structure using acetamiprid. Accordingly, acetamiprid adsorption on graphene oxide shows higher stability compared to its adsorption on graphite or multi-walled nanotubes.

**Table 1:** Graphene oxide, graphite, and multi-walled nanotubes docking energies.

Complex	∆E_ads_ (kcal/mol)
Graphite-Ace	+ 0.28
Graphene oxide-Ace	− 0.7
MWNT-Ace	+ 0.95

## Conclusion

The study examined Acetamiprid/graphene oxide, Acetamiprid/graphene and Acetamiprid/multi-walled nanotubes nanocomposites ability to comparatively remove and adsorb acetamiprid from aqueous solutions. Thorough analysis showed that graphene oxide layers contain a large variety of oxygen functionalities and Based on evidence obtained from experiments, the acetamiprid aqueous solution were independent from pH for the efficiency of their adsorption. Evidence also showed increased absorbent performance in adsorbing acetamiprid, according to which Acetamiprid/graphene, Acetamiprid/graphene oxide and Acetamiprid/multi-walled nanotubes had respective efficiencies of 97.5%, 92.5% and 95.3%. Based on the results obtained from experiments, the adsorption nature was in favor of Langmuir and Freundlich isotherm in the three adsorbents under study. According to data from equilibrium adsorption, the pseudo-second order had the greatest fitness, while the involvement of the chemisorption mechanism could be also observed. The studied adsorbents were increasingly stable and reproducible. Similar results were shown in theoretical and docking as well as empirical analyses.

## Data Availability

The datasets generated and/or analyzed during the current study are not publicly available due ethical cases, The authors have not received financial support from any public or private organizations and have paid all the costs themselves, but are available from the corresponding author on reasonable requests.
